# Dental Floss Prescription Pattern among the Dental Interns of Nepal

**DOI:** 10.31729/jnma.5133

**Published:** 2020-08-31

**Authors:** Nashib Pandey, Sushmit Koju, Anju Khapung, Sujaya Gupta, Deepa Aryai, Bhageshwar Dhami

**Affiliations:** 1 Department of Periodontics, Kantipur Dental College Teaching Hospital, Kathmandu, Nepal; 2 Department of Oral Pathology, Kantipur Dental College Teaching Hospital, Kathmandu, Nepal; 3 Department of Community Dentistry, College of Dental Sciences, Nepal Medical College, Kathmandu, Nepal; 4 Department of Periodontics and Oral Implantology, Kathmandu Medical College, Bhaktapur, Nepal

**Keywords:** *dental floss*, *interdental aids*, *oral hygiene*, *periodontitis*

## Abstract

**Introduction:**

Periodontal disease is regarded as one of the preventable diseases. It can be prevented through effective plaque control measures that require mechanical instrumentation with various surgical or non-surgical periodontal therapies as well as daily oral hygiene measures. Dental professionals must be competent enough to promote good oral health by educating patients with daily oral hygiene practice measures. In this regard, the study was designed to assess the knowledge, attitude, and practice behaviour for using as well as prescribing dental floss among the dental interns of Nepal.

**Methods:**

An online questionnaire consisting of two sections; the first comprised of the sociodemographic and professional aspects and the second consisted of questions related to knowledge, attitude, and practice regarding the use of dental floss, and its prescription was prepared using Google forms and the link was shared. The data were analysed in Statistical Package for Social Sciences (SPSS) version 20 software.

**Results:**

In this study, the participants were familiar with the dental floss, but many lacked awareness regarding its proper use. Ninety-nine (64.3%) of the participants personally used dental floss. Only six (3.9%) never prescribed it to their patients. Among those who prescribed, only 37 (25%) always demonstrated the techniques.

**Conclusions:**

The study indicated that many participants used dental floss, however, educating and recommending the patient about it was comparatively low. This emphasizes the need to increase the awareness and use of interdental aids among dental interns to provide good oral self-care practices for the patient.

## INTRODUCTION

Periodontitis is a ubiquitous disease affecting over 50% of the world's adult population and increases further with age.^1^ Severe periodontitis, a major cause of tooth loss^2^ is caused by the accumulation of a plaque biofilm at and below the gingival margin. Plaque removal and/ or control is therefore fundamentally important for the prevention of periodontal diseases.

In addition to brushing and mouth rinses, American Dental Association (ADA) recommends the use of dental floss as one of the three key hygiene practices.^3^ Promoting good oral health by educating patients with daily oral hygiene practice measures are vital for achieving optimal effectiveness and preventing trauma. The future dentists should have adequate knowledge regarding its use, proper prescription as well as demonstration to their patient.

Hence, the present study was designed to assess the knowledge, attitude, and practice behaviour for using as well as prescribing dental floss among the dental interns of Nepal.

## METHODS

A cross-sectional descriptive study using an online questionnaire was conducted among dental interns of Nepal. Interns were selected as they are on the verge of their independent dental practice which affects the patients directly in their oral health practice and maintenance. The ethical approval was obtained from the Institutional Review Committee (IRC), Kantipur Dental College and Teaching Hospital (KDCH). Data collection was carried out for four months duration (from March to June 2020). Sample size of 138 was calculated by utilising the following formula for finite population:

Sample size,

$$\text{n}=\frac{\frac{{\text{Z}}^{\text{2}}\text{ p(1}-\text{p)}}{{\text{e}}^{\text{2}}}}{1+\frac{{\text{Z}}^{\text{2}}\text{ p(1}-\text{p)}}{{\text{e}}^{\text{2}}\text{N}}}$$

Z = 1.96 at confidence level = 95%; p = 0.85 (85%)^4^ with Margin of error (e) = 0.05 (5%); N = 456, total number of dental interns of Nepal obtained by asking the dental intern representative from 12 dental colleges of Nepal.

An online questionnaire after reviewing the pertinent literature^4–8^ was prepared using Google forms and a link was created. The questionnaire was pretested among dental interns of KDCH who were not included in the study sample. The reliability of the questionnaire was tested by Cronbach alpha and the value was found to 0.71. Suggestions for improvement in the questionnaires were incorporated from subject experts and modified accordingly. Link to the questionnaire and a cover letter along with an informed consent form were distributed to the dental interns' representative of each dental college of Nepal. This questionnaire consisted of two sections: the first comprised of the socio-demographic and professional aspects and the second section consisted of questions related to knowledge, attitude, and practice regarding the use of dental floss and its prescription. After the completion of questionnaire, the study participants were also provided with the resources related to the use and prescription of dental floss. The data from Google forms were entered in Microsoft Excel and analysed using descriptive statistics and presented as frequency and percentages in Statistical Package for Social Sciences (SPSS) version 20 software.

## RESULTS

**Figure 1.**
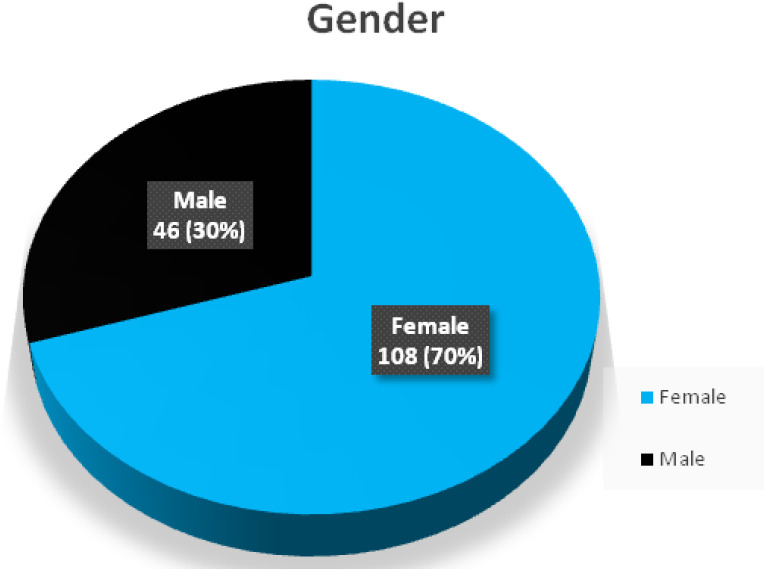
Gender wise distribution of the respondents.

Although the calculated sample size was 138, a total number of 154 dental interns all over the country responded to the questionnaire whose responses were included for the analysis. The mean age of the study participants was 24.21±1.24 years. The gender wised distribution of study participants is given in Figure 1. Majority of the participants of the study lacked proper dental floss prescription related knowledge. Distribution of study participants according to knowledge, and attitude, of using as well as prescribing dental floss are depicted in Table 1 and Table 2 respectively.

**Table 1:** Participants' knowledge regarding dental floss.

Questions	Responses	Males n (%)	Females n (%)	Total n (%)
Dental foss is prescribed for	Open contacts	2 (4.3)	9 (8.3)	11 (7.1)
	Tight contacts	29 (63)	60 (55.6)	89 (57.8)
	Both situations	15 (32.6)	39 (36.1)	54 (35.1)
Dental floss is prescribed for removing	Interdental plaque	38 (82.6)	75 (69.4)	113 (73.4)
	Impacted food particles	33 (71.7)	88 (81.5)	121 (78.6)
	Material alba	8 (17.4)	18 (16.7)	26 (16.9)
	Debris	20 (43.5)	35 (32.4)	55 (35.7)
Dental floss should be prescribed	Before brushing	12 (26.1)	21 (19.4)	33 (21.4)
	After brushing	26 (56.5)	65 (60.2)	91 (59.1)
	Both situations	8 (17.4)	22 (20.4)	30 (19.5)
Use of dental floss would injure interdental gingiva	Yes	17 (37)	31 (28.7)	48 (31.2)
	No	15 (32.6)	46 (42.6)	61 (39.6)
	I don't know	14 (30.4)	31 (28.7)	45 (29.2)
Use of dental floss increases the spacing between teeth	Yes	2 (4.3)	10 (9.3)	12 (7.8)
	No	36 (78.3	79 (73.1)	115 (74.7)
	I don't know	8 (17.4)	19 (17.6)	27 (17.5)
Use of dental floss causes gingival bleeding	Yes	11 (23.9)	26 (24.1)	37 (24)
	No	22 (47.8)	51 (47.2)	73 (47.4)
	I don't know	13 (28.3)	31 (28.7)	44 (28.6)
Dental floss fraying results during flossing of proximal restoration/ crown and bridges	Yes	23 (50)	61 (56.5)	84 (54.5)
	No	8 (17.4)	22 (20.4)	30 (19.5)
	I don't know	15 (32.6)	25 (23.1)	40 (26)

**Table 2:** Participants' attitude towards dental floss.

Questions	Responses	Males n (%)	Females n (%)	Total n (%)
Dental floss can be reused after rinsing with water	Agree	5 (10.9)	6 (5.6)	11 (7.1)
	Disagree	38 (82.6)	91 (84.3)	129 (83.8)
	Neutral	3 (6.5)	11 (10.1)	14 (9.1)
Intraoral swishing can be used as an alternative/ adjunct to dental flossing	Agree	10 (21.7)	26 (24.1)	36 (23.4)
	Disagree	27 (58.7)	61 (56.5)	88 (57.1)
	Neutral	9 (19.6)	21 (19.4)	30 (19.5)
Dental floss shouldn't be used in sites with proximal restoration/ crown and bridges	Agree	4 (8.7)	7 (6.5)	11 (7.2)
	Disagree	36 (78.3)	92 (85.2)	128 (83.1)
	Neutral	6 (13)	9 (8.3)	15 (9.7)
Dental floss should be used once daily	Agree	37 (80.4)	79 (73.2)	116 (75.4)
	Disagree	3 (6.5)	16 (14.8)	19 (12.3)
	Neutral	6 (13.1)	13 (12)	19 (12.3)
There is no added advantage in prescribing dental floss to patients using sulcular brushing technique	Agree	0 (0)	4 (3.7)	4 (2.6)
	Disagree	37 (80.4)	70 (64.8)	107 (69.5)
	Neutral	9 (19.6)	34 (31.5)	43 (27.9)

Regarding the practice, 99 (64.3 %) of them have been using dental floss personally and 148 (96%) are prescribing it. Among the prescribers, only 37 (25%) always demonstrated the techniques. Frequency distribution of study participants according to gender regarding their practice of using as well as prescribing dental loss is depicted in (Table 3).

**Table 3:** Participants' practice for the use and prescription of dental floss.

Questions	Responses	Males n (%)	Females n (%)	Total n (%)
Do you personally use dental floss?	Yes	24 (52.2)	75 (69.4)	99 (64.3)
	No	22 (47.8)	33 (30.6)	55 (35.7)
Have you ever prescribed dental floss to your patients?	Always	6 (13)	21 (19.4)	27 (17.5)
	Sometimes	35 (76.1)	86 (79.6)	121 (78.6)
	Never	5 (10.9)	1 (1)	6 (3.9)
Do you demonstrate the technique of using dental floss to your patients?	Always	8 (19.5)	29 (27.1)	37 (25)
	Sometimes	21 (51.3)	32 (29.9)	53 (35.8)
	Never	1 (2.4)	2 (1.9)	3 (2.1)
	Depend upon patients	11 (26.8)	44 (41.1)	55 (37.1)
What do you prefer to demonstrate the technique of using dental floss to your patients?	Your own mouth	3 (7.3)	4 (3.7)	7 (4.7)
	Patients own mouth	4 (9.8)	17 (15.9)	21(14.2)
	Typhodont model	34 (82.9)	86 (80.4)	120 (81.1)

## DISCUSSION

Dental floss has been used since prehistoric times. Father of oral hygiene, Levi Spear Parmly, a dentist from New Orleans, has been credited for inventing the modern dental floss.^9^ In 1815, he recommended flossing with a piece of silk thread, through the interstices of the teeth, between their necks and the arches of the gum, to dislodge that irritating matter which no brush can remove and which is the real source of disease. Since then, humble silk thread has undergone tremendous transformation in modern times.^9,10^ Although different varieties of floss products have been developed, any floss product in combination with a manual toothbrush can remove plaque significantly better than the toothbrush alone.^11–12^

Interdental cleaning aids should be professionally taught not only to patients with gingival inflammation but also to those with healthy gingiva. Specific prescription for dental floss is recommended in cases with healthy sites where attachment loss is not evident and trauma and/or increased space may result due to the improper use of interdental brushes.^2^ Professional instructions for using dental floss at home have much impact in preventing periodontal disease. Hence, the knowledge, attitude, and practices, for using as well as prescribing dental floss among the future dental practitioners who are about to start their professional career to identify the deficiencies are essential. This can be helpful to plan corrective measures. The present study assessed the dental floss prescription-related knowledge, attitude, practice among dental interns of Nepal, which can be utilised in the reformation of oral health related topics of dental programs of Nepal.

In the current study, 99 (64.3%) of the dental interns surveyed have been using dental floss personally. Various studies conducted across the globe have reported with the percentages of dentists using dental floss with 22% in India,^4^ 23.4% in Japan,^5^ and 56.3% in the US.^6^ The current oral hygiene practice of the prescribers may affect the prescription pattern.^4^ A maximum number of dental interns using dental floss as their interdental cleaning aid could have motivated them in prescribing it. It was observed that more than 96% of the dental interns had prescribed dental floss to their patients. A study conducted among the Indian dentists reported 63.9% of dentists prescribed floss routinely to their patients.^4^ Although, low in number, the reason for not being involved in prescribing dental floss in the start of their clinical practice might be they have not been posted in the respective department where the floss is to be prescribed. Only 37 (25%) respondents “always” demonstrated the technique of flossing to the patients in our study, which can be attributed to the lack of demonstration of dental flossing procedure at the dental schools by their teachers as reported by Nakamura et al.^5^

In this study, more than 75% of the participants agreed that dental floss should be used once daily. In a study done among different health care professional in the United States, it was found that persons flossing less than once a day were as likely to have periodontitis as those who flossed daily (OR = 1.16, 95% CI: 0.63 to 2.13) after controlling for the profession, age, gender, smoking, diabetes, coronary heart disease, history of periodontal surgery, and the number of teeth present.^6^ Another study conducted among 44 dental students of School of Dentistry, Kerman, Iran, have come with a conclusion that if a person with normal periodontal tissues uses the toothbrush and dental foss properly, using dental floss in every other day is sufficient to maintain the gingival health.^7^

Passing dental floss between contact areas is the most widely used method to evaluate interproximal contacts by the operator,^13^ but it can be prescribed for both open as well as tight contact areas. Although the interdental brush is prescribed for open contact areas, dental floss can also be prescribed if a proper technique has been demonstrated based on the patients' teeth alignment in the arch. Just prescribing dental floss is not enough; it is also essential to demonstrate correct flossing technique, as incorrect flossing may result in cervical abrasion of the teeth and angular alveolar bone loss.^14,15^ Several diverse factors significantly influence the way students perceive and experience their education. The improvements can be made with increased formative assessment and self-assessment opportunities, collaborative learning, familiarisation with and increased implementation of information and communication technology applications, early clinical exposure.^16^

In the current study, the majority of the participants 91 (59.1%) believed that dental floss should be prescribed after brushing. However, in a Randomised Controlled Clinical Trial, researchers have found that the amount of plaque between the teeth and in the mouth overall was significantly reduced when participants used the floss before brushing. They contend that as flossing loosens bacteria and debris from between the teeth, brushing afterward (when the mouth is rinsed with water) further clears the mouth of these particles.^8^

Various myths related to dental floss among the patients like it increases bleeding gums, and spacing between teeth was not prevalent in the majority of the study population. We had asked the participants about intraoral swishing. “Can, intraoral swishing be used as an alternative/ adjunct to dental flossing?” The majority (88, 57%) of the participants didn't agree on this note. The much-overlooked procedure of swishing of water after the consumption of foods and soft drinks can be a safe, economic and easy means of improving oral hygiene in addition to tooth brushing.^17^ Swishing 20-30 ml of water after eating food or consumption of soft drinks and also between meals for two to five minutes can be of help in the removal of loosened food particles, dead cells and mucous from the oral cavity.^18^ Warming the water and adding a pinch of table salt reduces inflammation and can be microbicidal because of its high osmolality.^19^ Studies have even revealed that oral irrigation is an effective alternative to manual tooth brushing and dental flossing for reducing bleeding and gingival inflammation.^20^ Dental waterjets in the form of commercially available water flosser (e.g., Waterpik) and mouthwashes are expensive but vigorous water swishing using movement of the lips, tongue, cheeks can be a beneficial alternative for goodoral hygiene.^17^ Various literature supporting the efficacy of commercially available water flosser can be found abundantly^21–25^ but very few have mentioned about the role of vigorous water swishing using the movement of the lips, tongue, cheeks. This may be because of the commercial interest of the manufacturers. The low number of evidence for Water swishing has been reflected in the knowledge of dental interns of Nepal as well. Future research can be directed towards comparing the efficacy of vigorous water swishing using the movement of the lips, tongue, cheeks with other interdental aids.

Checking practical demonstrations along with a self-reported questionnaire rather than just a questionnaire could have given us with the actual practice status of dental interns. Initially, during the proposal development, the study was designed to check the steps of flossing in a typhodont model along with the questionnaire. But, because of the COVID-19 lockdown imposed by the government of Nepal during the data collection period, we faced difficulty in reaching each dental intern in person. So the demonstration part was not carried and IRC was informed about this. Future studies related to dental floss practice can be conducted by incorporating the demonstration part as well. The authors would like to recommend that similar study can be conducted among other dental professionals as well as the general population to understand the need and practices of dental floss.

## CONCLUSIONS

The results obtained from the present study indicate the necessity of motivating the students and interns to follow self-oral hygiene practices and also educate the patients regarding the same. Though many participants use dental floss, it was seen that educating and recommending the patient about it was comparatively low. This emphasizes the need to increase the awareness and use of aids among dental interns to provide good oral self-care practices for the patient. Patient education through practical demonstration of dental floss use should be made compulsory to the dental interns so that they can increase their confidence level in demonstrating it to the patients.
